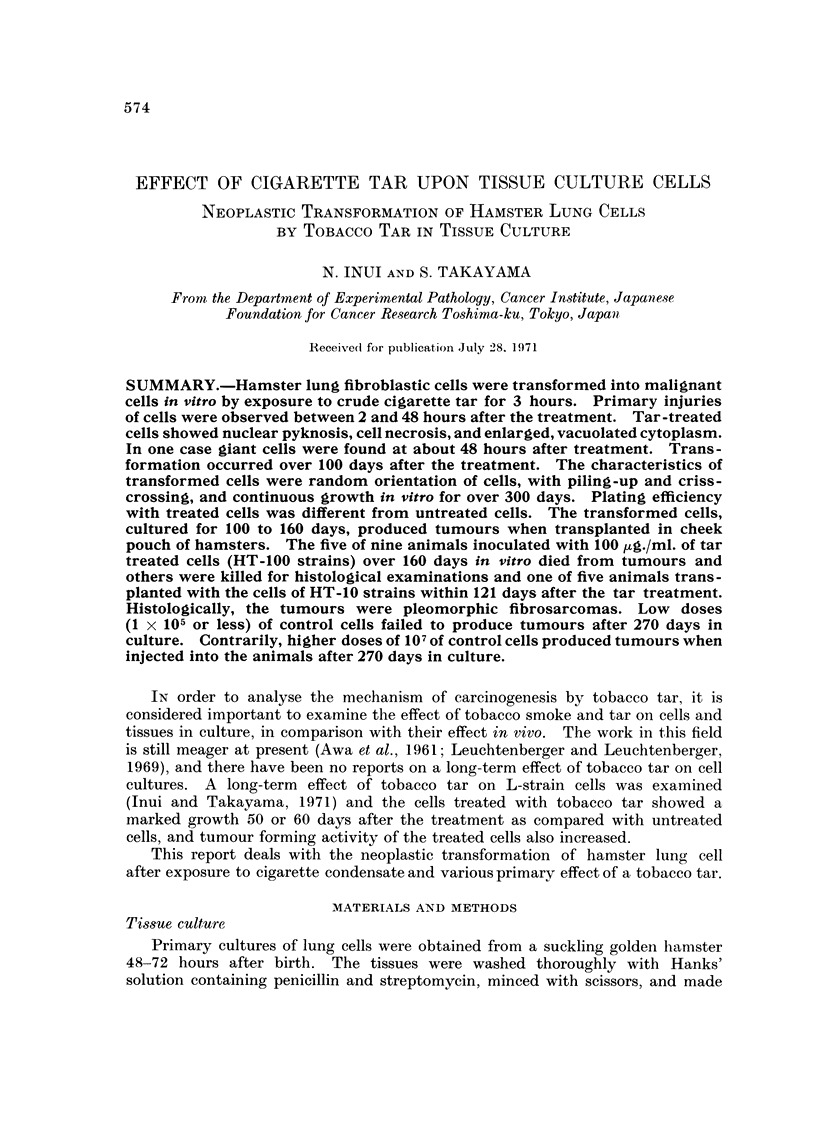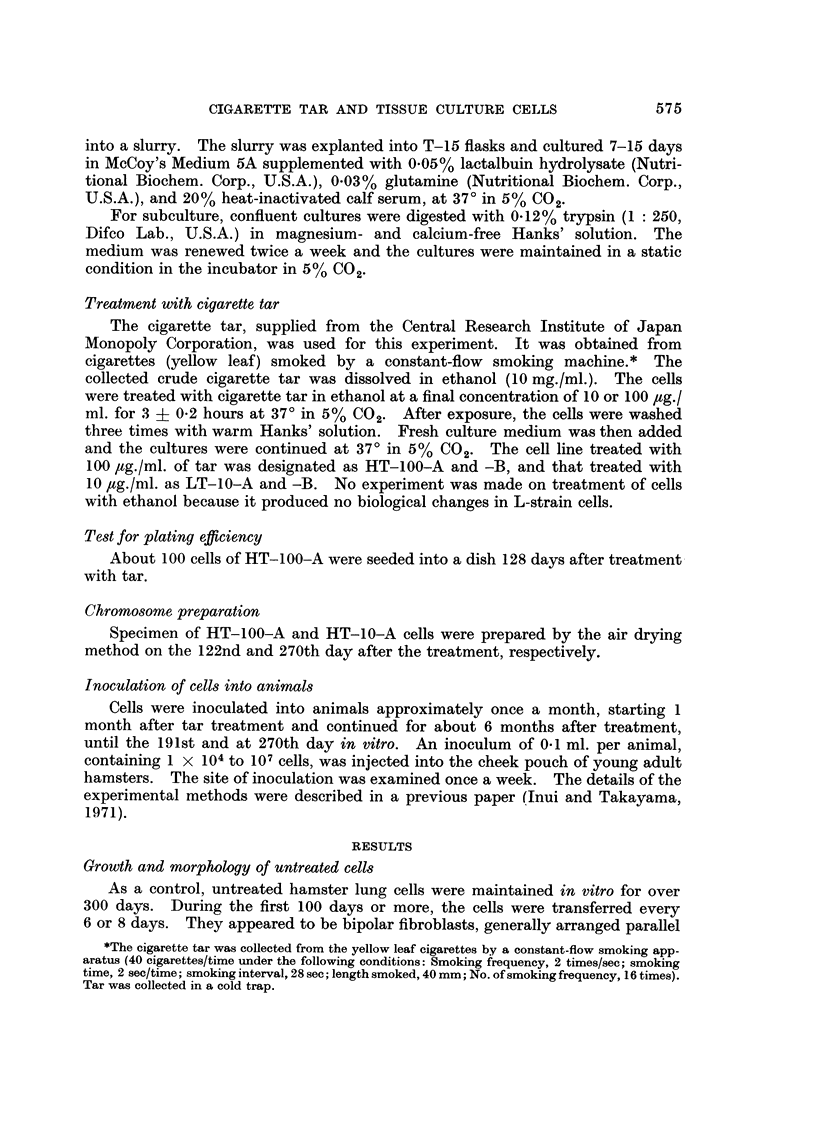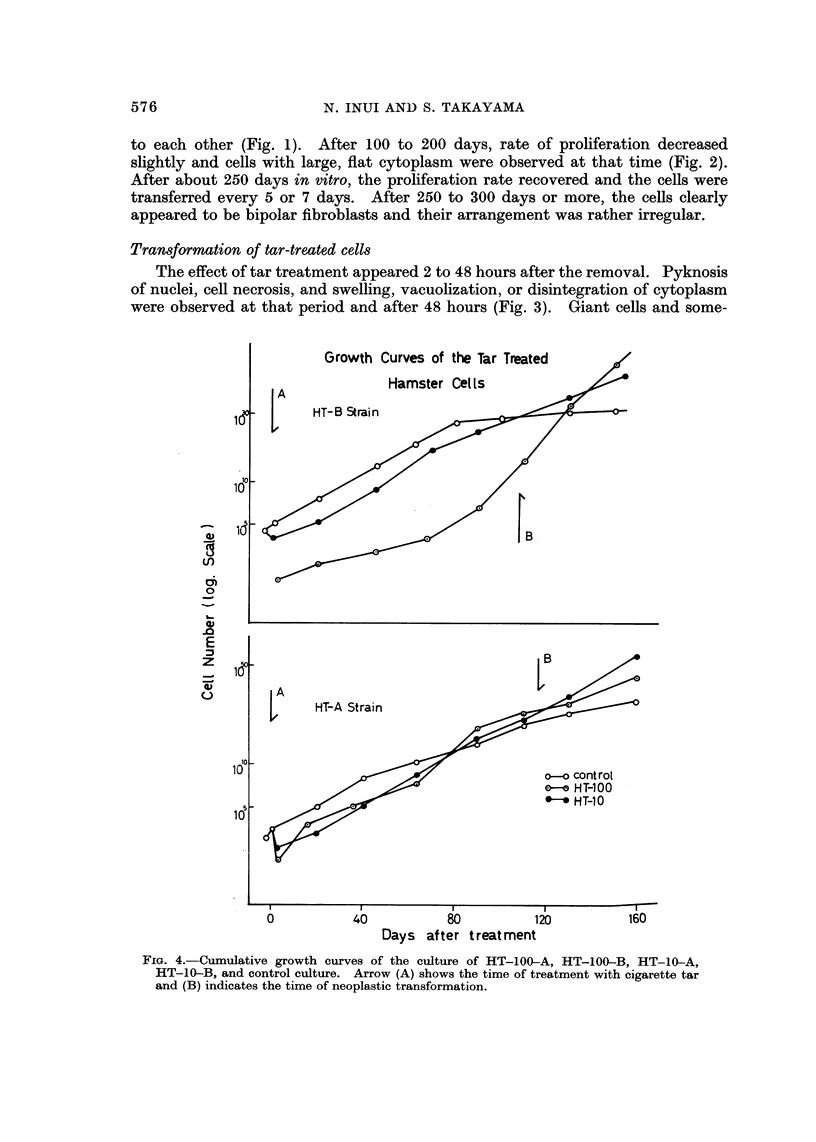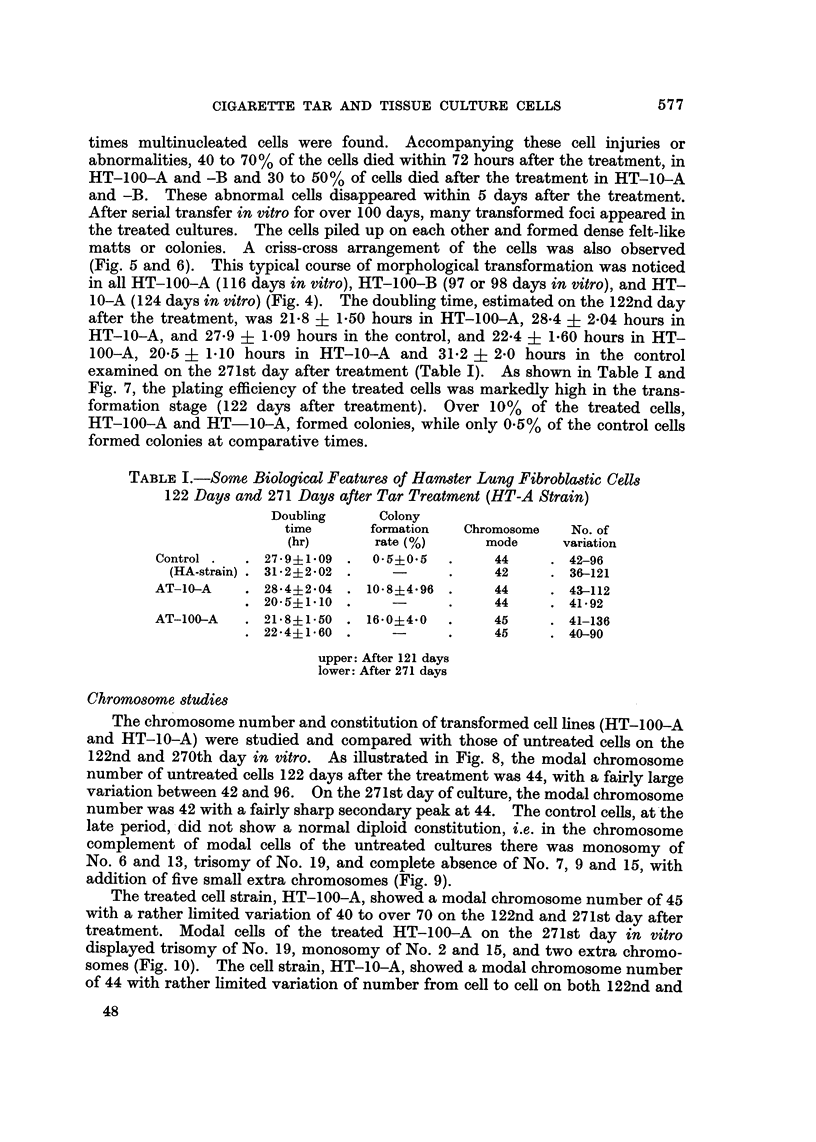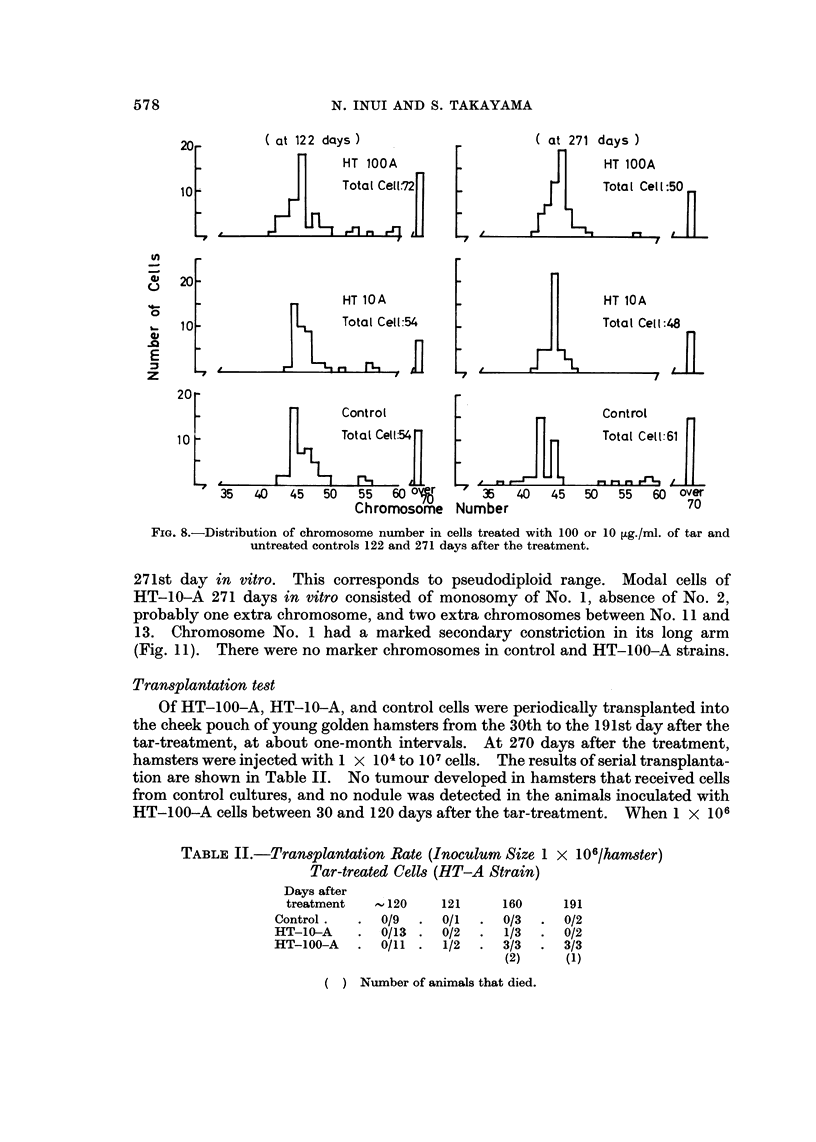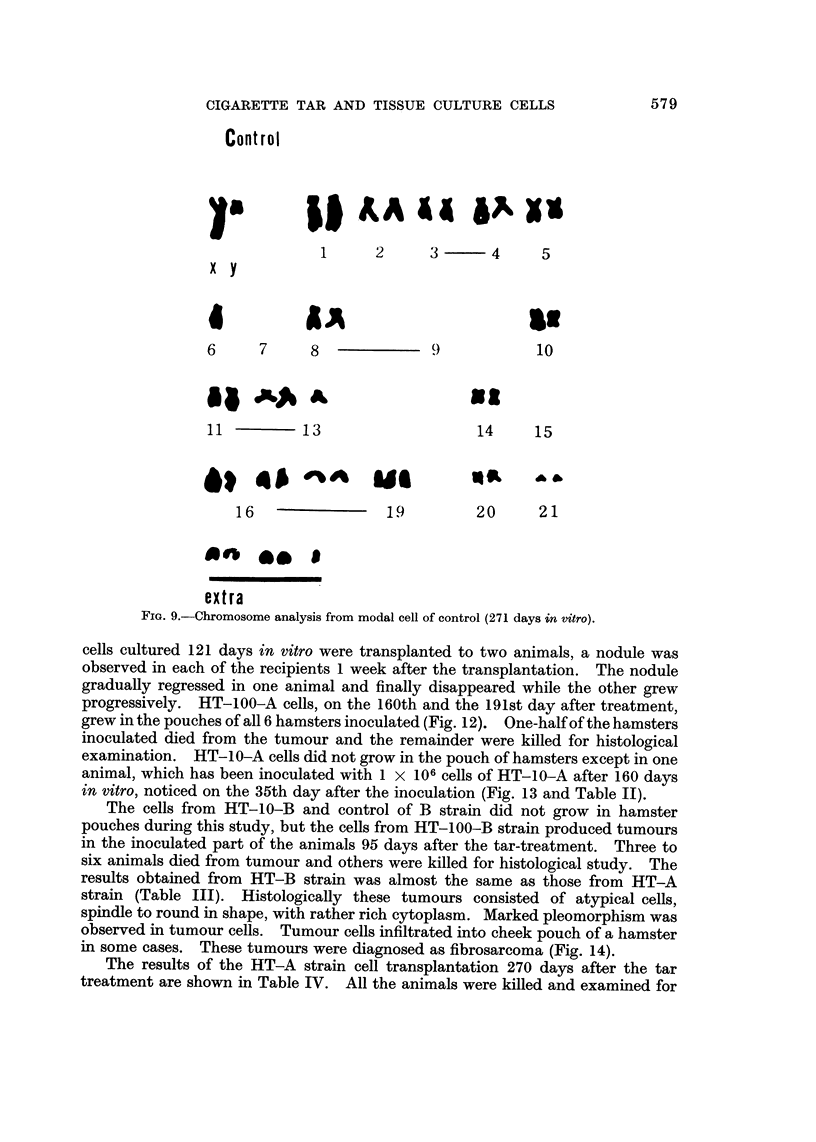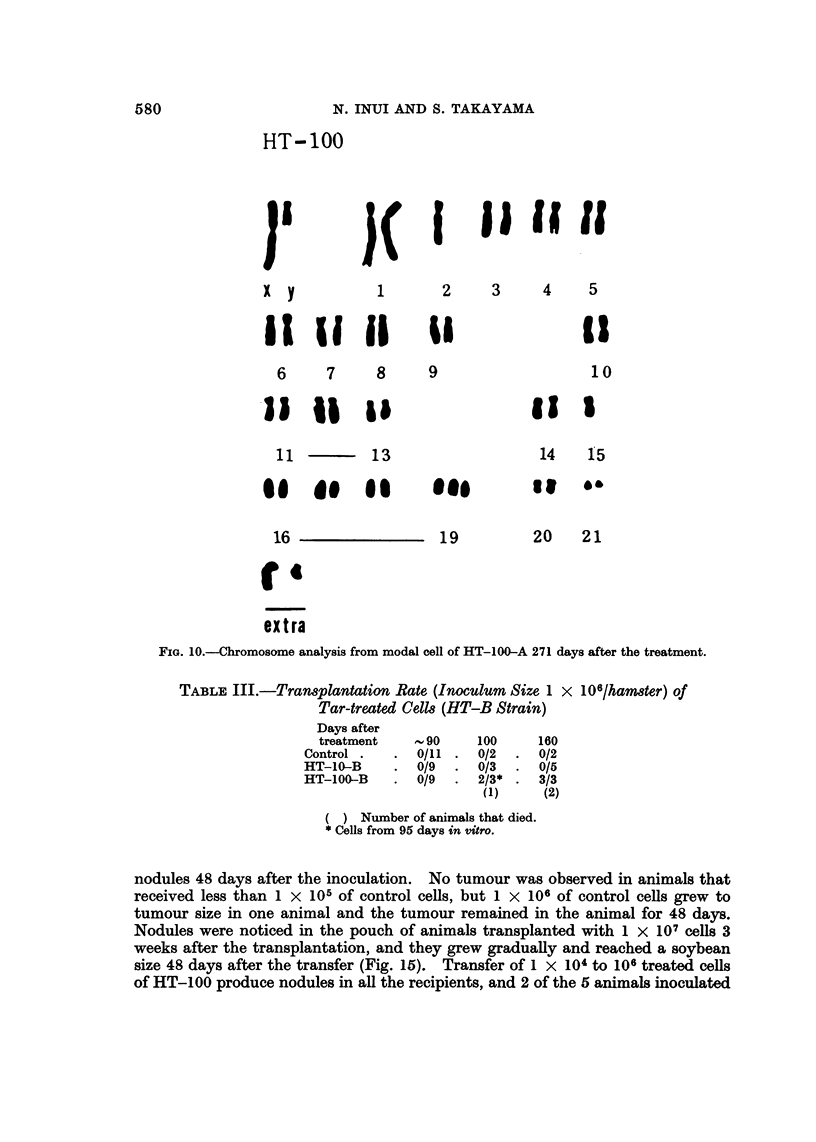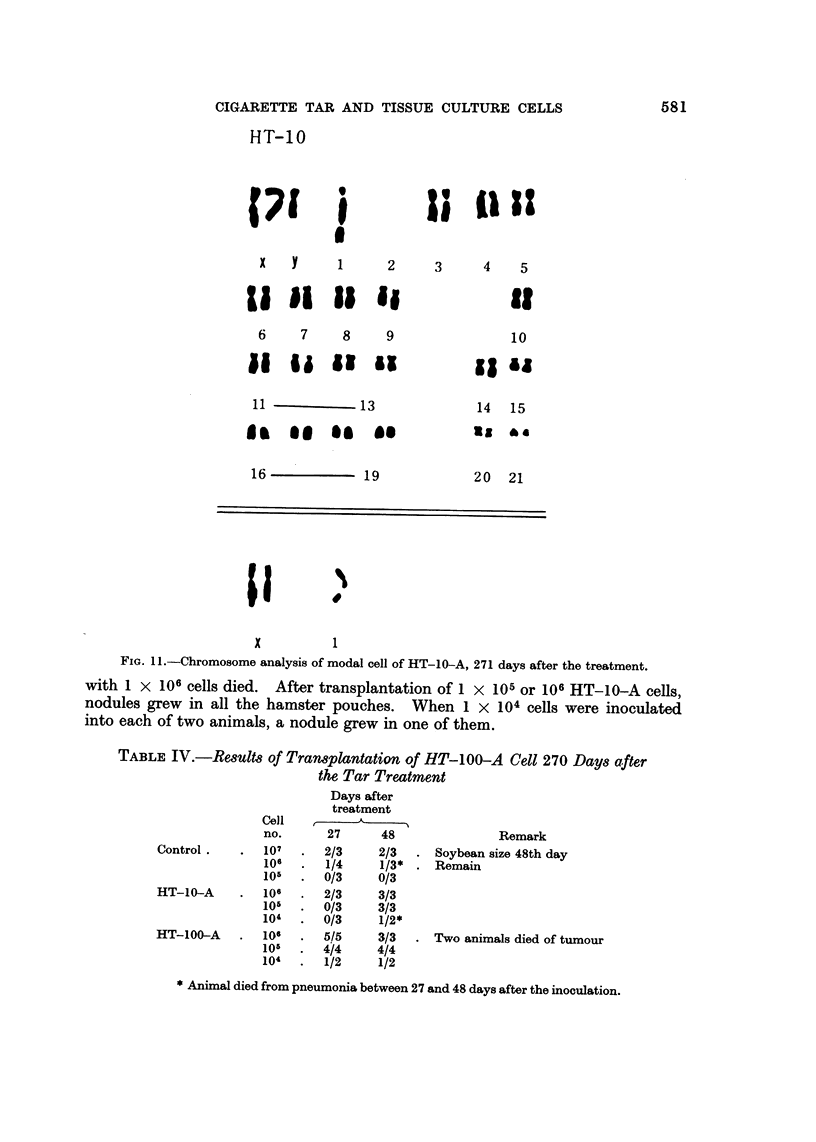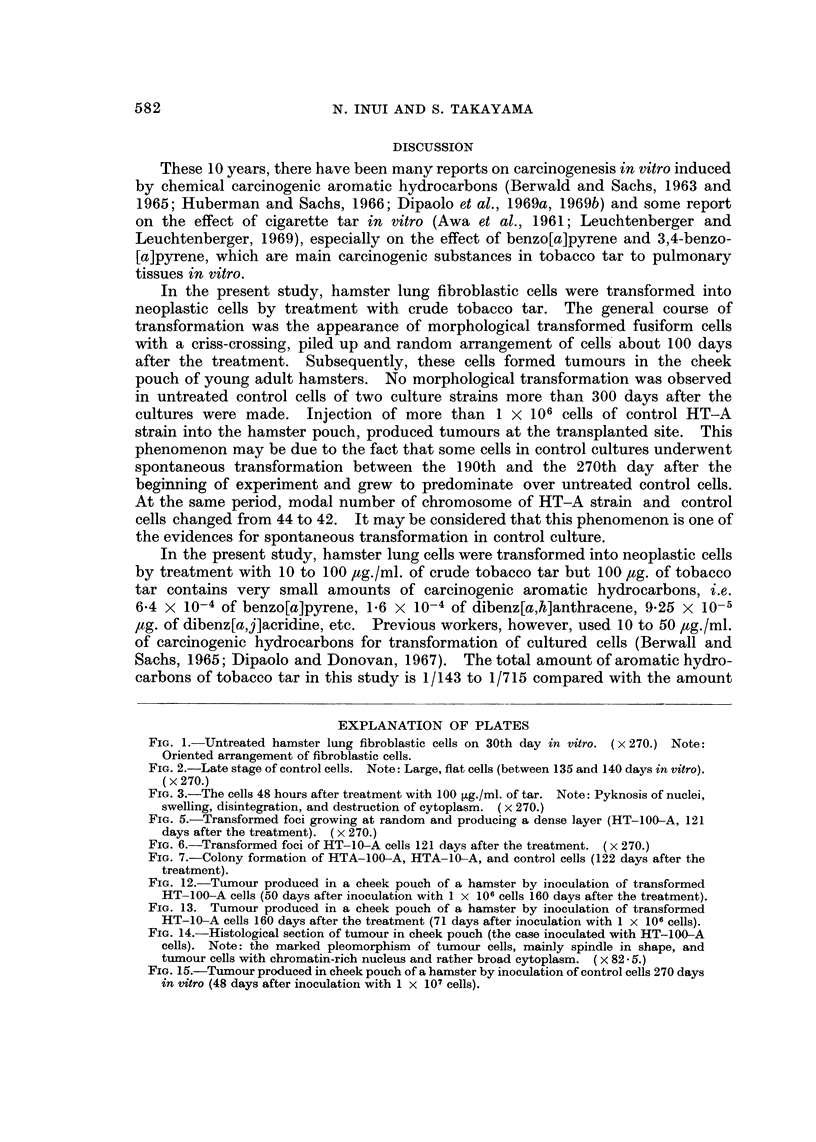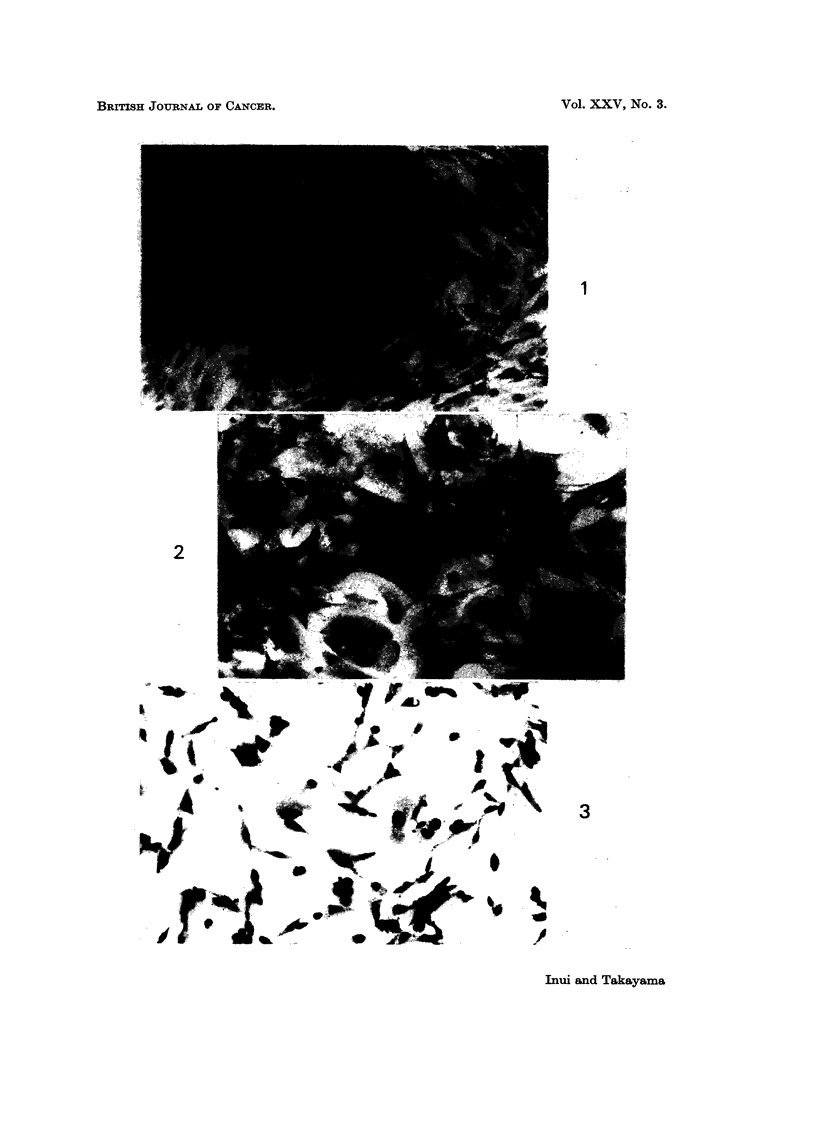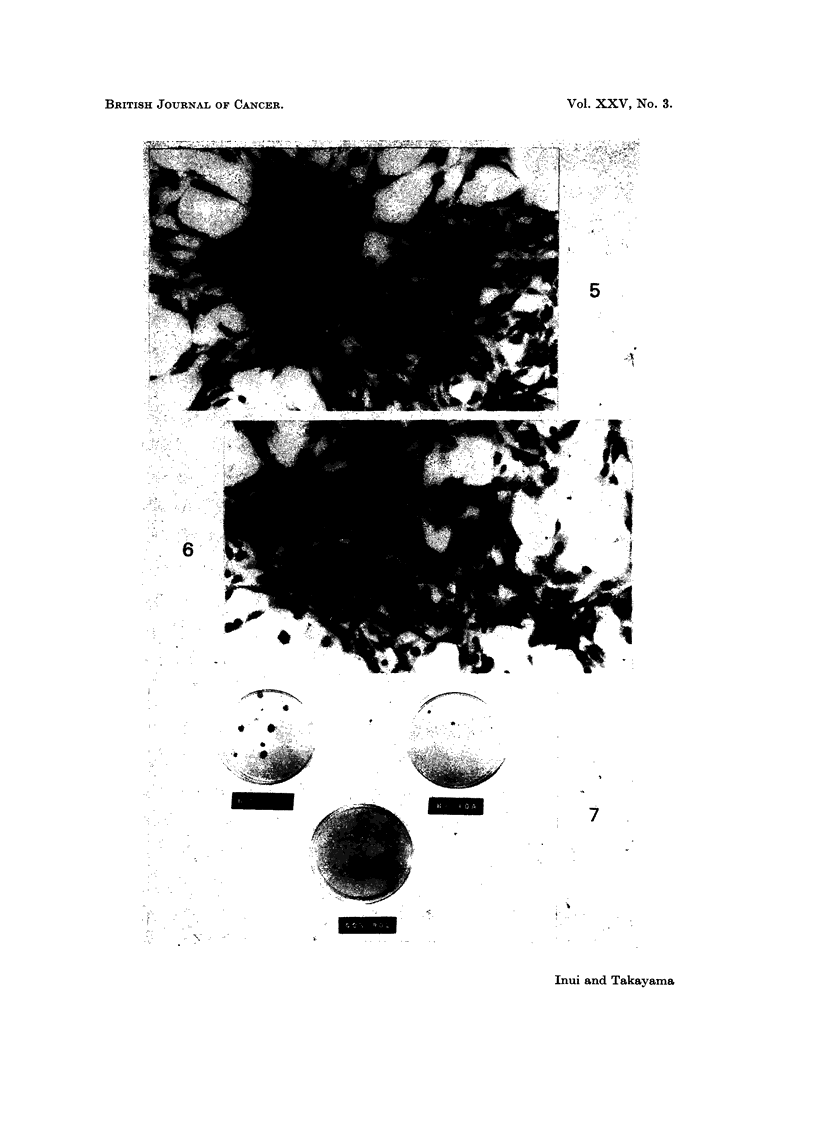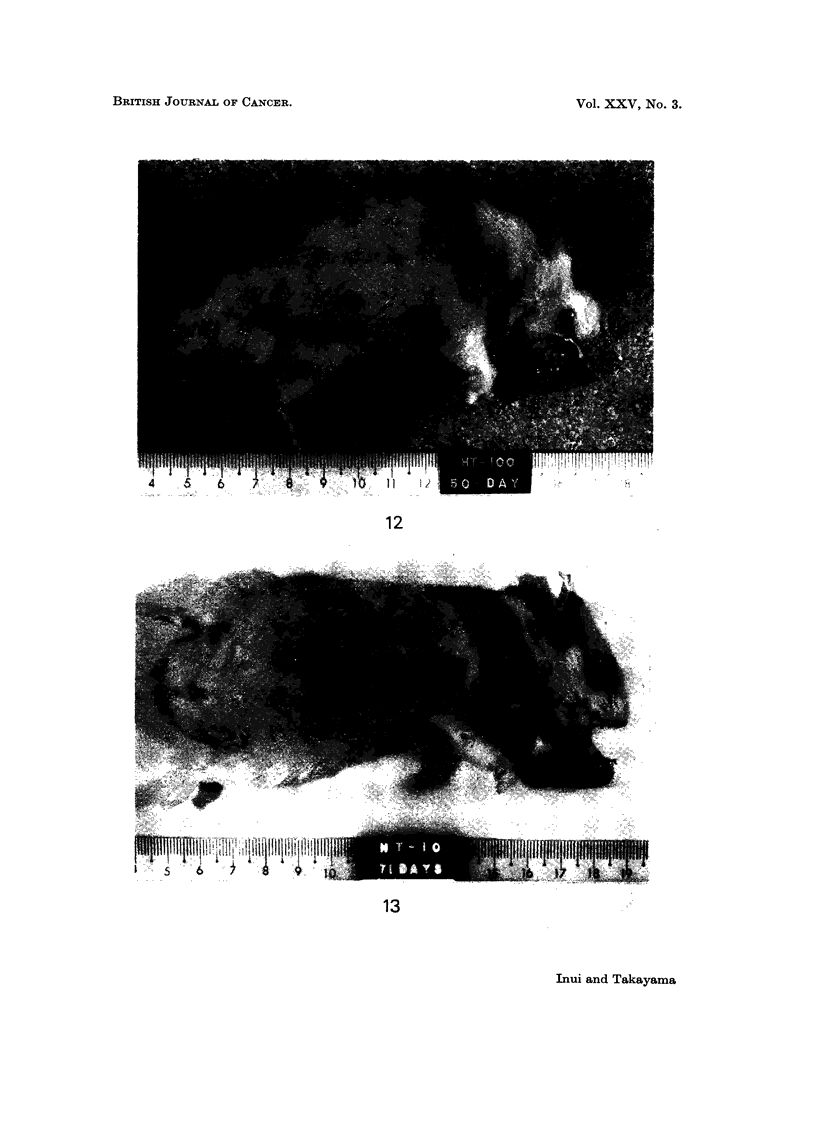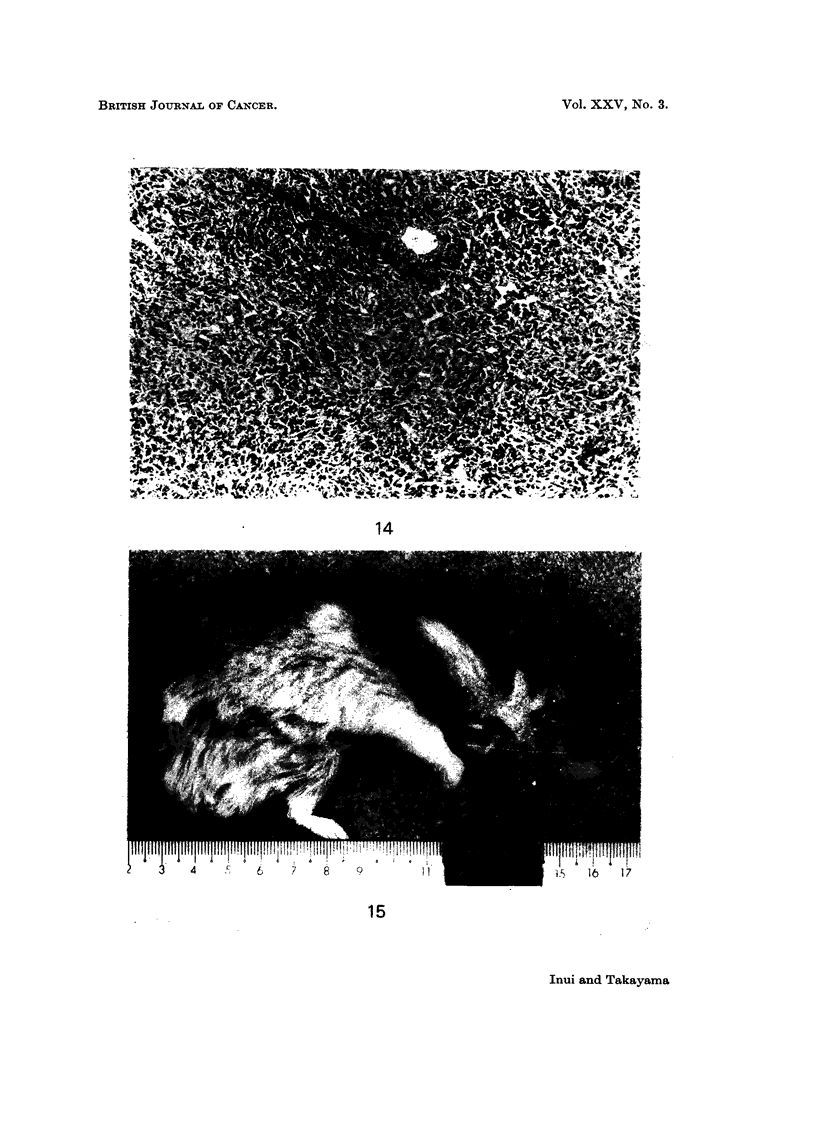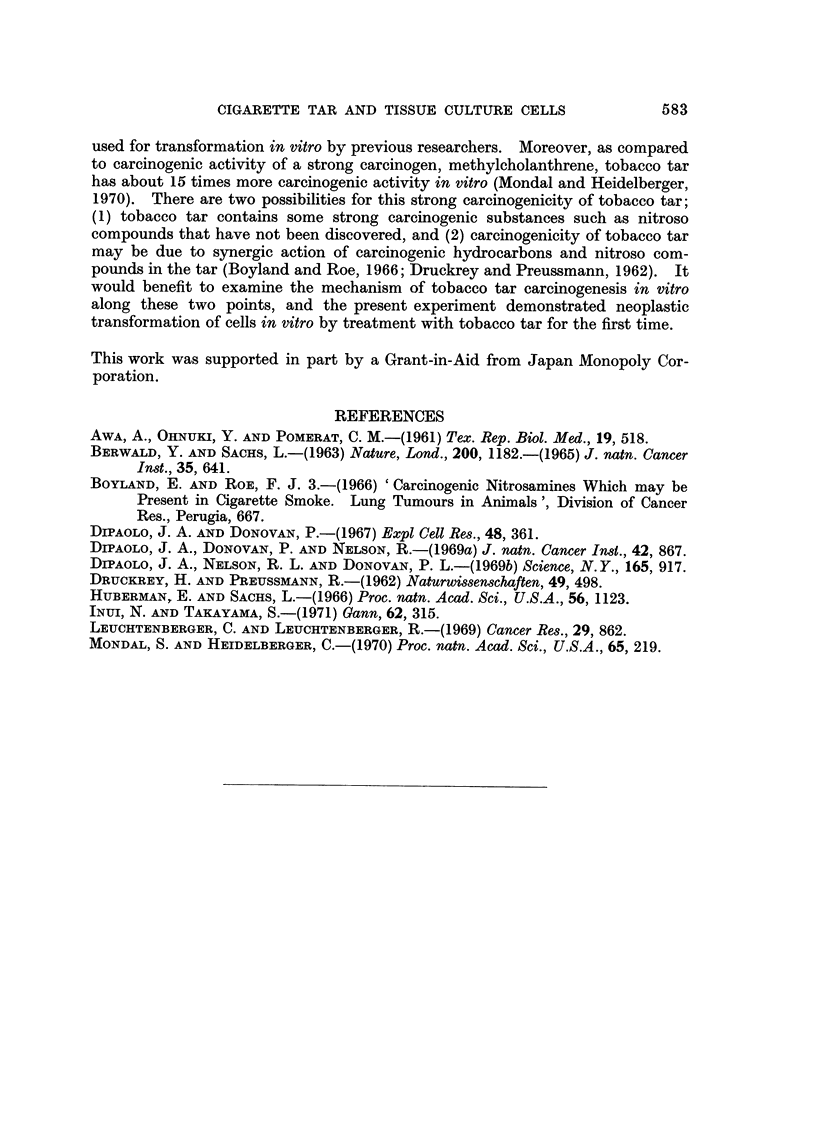# Effect of Cigarette Tar upon Tissue Culture Cells

**DOI:** 10.1038/bjc.1971.73

**Published:** 1971-09

**Authors:** N. Inui, S. Takayama

## Abstract

**Images:**


### EFFECT OF CIGARETTE TAR UPON TISSUE CULTURE CELLS

### NEOPLASTIC TRANSFORMATION OF HAMSTER LUNG CELLS BY TOBACCO TAR IN TISSUE CULTURE

N. INUI AND S. TAKAYAMA

*From the Department of Experimental Pathology, Cancer Institute, Japanese Foundation for Cancer Research Toshima-ku, Tokyo, Japan*

Received for publication July 28, 1971

**SUMMARY.—Hamster lung fibroblastic cells were transformed into malignant cells *in vitro* by exposure to crude cigarette tar for 3 hours. Primary injuries of cells were observed between 2 and 48 hours after the treatment. Tar-treated cells showed nuclear pyknosis, cell necrosis, and enlarged, vacuolated cytoplasm. In one case giant cells were found at about 48 hours after treatment. Transformation occurred over 100 days after the treatment. The characteristics of transformed cells were random orientation of cells, with piling-up and criss-crossing, and continuous growth *in vitro* for over 300 days. Plating efficiency with treated cells was different from untreated cells. The transformed cells, cultured for 100 to 160 days, produced tumours when transplanted in cheek pouch of hamsters. The five of nine animals inoculated with 100 $\mu$g./ml. of tar treated cells (HT-100 strains) over 160 days *in vitro* died from tumours and others were killed for histological examinations and one of five animals transplanted with the cells of HT-10 strains within 121 days after the tar treatment. Histologically, the tumours were pleomorphic fibrosarcomas. Low doses ($1 \times 10^5$ or less) of control cells failed to produce tumours after 270 days in culture. Contrarily, higher doses of $10^7$ of control cells produced tumours when injected into the animals after 270 days in culture.**

IN order to analyse the mechanism of carcinogenesis by tobacco tar, it is considered important to examine the effect of tobacco smoke and tar on cells and tissues in culture, in comparison with their effect *in vivo*. The work in this field is still meager at present (Awa *et al.*, 1961; Leuchtenberger and Leuchtenberger, 1969), and there have been no reports on a long-term effect of tobacco tar on cell cultures. A long-term effect of tobacco tar on L-strain cells was examined (Inui and Takayama, 1971) and the cells treated with tobacco tar showed a marked growth 50 or 60 days after the treatment as compared with untreated cells, and tumour forming activity of the treated cells also increased.

This report deals with the neoplastic transformation of hamster lung cell after exposure to cigarette condensate and various primary effect of a tobacco tar.

### MATERIALS AND METHODS

*Tissue culture*

Primary cultures of lung cells were obtained from a suckling golden hamster 48–72 hours after birth. The tissues were washed thoroughly with Hanks' solution containing penicillin and streptomycin, minced with scissors, and made into a slurry. The slurry was explanted into T–15 flasks and cultured 7–15 days in McCoy's Medium 5A supplemented with 0·05% lactalbuin hydrolysate (Nutritional Biochem. Corp., U.S.A.), 0·03% glutamine (Nutritional Biochem. Corp., U.S.A.), and 20% heat-inactivated calf serum, at 37° in 5% $CO_2$.

For subculture, confluent cultures were digested with 0·12% trypsin (1 : 250, Difco Lab., U.S.A.) in magnesium- and calcium-free Hanks' solution. The medium was renewed twice a week and the cultures were maintained in a static condition in the incubator in 5% $CO_2$.

*Treatment with cigarette tar*

The cigarette tar, supplied from the Central Research Institute of Japan Monopoly Corporation, was used for this experiment. It was obtained from cigarettes (yellow leaf) smoked by a constant-flow smoking machine.* The collected crude cigarette tar was dissolved in ethanol (10 mg./ml.). The cells were treated with cigarette tar in ethanol at a final concentration of 10 or 100 $\mu$g./ml. for 3 $\pm$ 0·2 hours at 37° in 5% $CO_2$. After exposure, the cells were washed three times with warm Hanks' solution. Fresh culture medium was then added and the cultures were continued at 37° in 5% $CO_2$. The cell line treated with 100 $\mu$g./ml. of tar was designated as HT–100–A and –B, and that treated with 10 $\mu$g./ml. as LT–10–A and –B. No experiment was made on treatment of cells with ethanol because it produced no biological changes in L-strain cells.

*Test for plating efficiency*

About 100 cells of HT–100–A were seeded into a dish 128 days after treatment with tar.

*Chromosome preparation*

Specimen of HT–100–A and HT–10–A cells were prepared by the air drying method on the 122nd and 270th day after the treatment, respectively.

*Inoculation of cells into animals*

Cells were inoculated into animals approximately once a month, starting 1 month after tar treatment and continued for about 6 months after treatment, until the 191st and at 270th day *in vitro*. An inoculum of 0·1 ml. per animal, containing $1 \times 10^4$ to $10^7$ cells, was injected into the cheek pouch of young adult hamsters. The site of inoculation was examined once a week. The details of the experimental methods were described in a previous paper (Inui and Takayama, 1971).

### RESULTS

*Growth and morphology of untreated cells*

As a control, untreated hamster lung cells were maintained *in vitro* for over 300 days. During the first 100 days or more, the cells were transferred every 6 or 8 days. They appeared to be bipolar fibroblasts, generally arranged parallel

*The cigarette tar was collected from the yellow leaf cigarettes by a constant-flow smoking apparatus (40 cigarettes/time under the following conditions: Smoking frequency, 2 times/sec; smoking time, 2 sec/time; smoking interval, 28 sec; length smoked, 40 mm; No. of smoking frequency, 16 times). Tar was collected in a cold trap.

to each other (Fig. 1). After 100 to 200 days, rate of proliferation decreased slightly and cells with large, flat cytoplasm were observed at that time (Fig. 2). After about 250 days *in vitro*, the proliferation rate recovered and the cells were transferred every 5 or 7 days. After 250 to 300 days or more, the cells clearly appeared to be bipolar fibroblasts and their arrangement was rather irregular.

*Transformation of tar-treated cells*

The effect of tar treatment appeared 2 to 48 hours after the removal. Pyknosis of nuclei, cell necrosis, and swelling, vacuolization, or disintegration of cytoplasm were observed at that period and after 48 hours (Fig. 3). Giant cells and some-

FIG. 4.—Cumulative growth curves of the culture of HT-100-A, HT-100-B, HT-10-A, HT-10-B, and control culture. Arrow (A) shows the time of treatment with cigarette tar and (B) indicates the time of neoplastic transformation.

times multinucleated cells were found. Accompanying these cell injuries or abnormalities, 40 to 70% of the cells died within 72 hours after the treatment, in HT–100–A and –B and 30 to 50% of cells died after the treatment in HT–10–A and –B. These abnormal cells disappeared within 5 days after the treatment. After serial transfer *in vitro* for over 100 days, many transformed foci appeared in the treated cultures. The cells piled up on each other and formed dense felt-like matts or colonies. A criss-cross arrangement of the cells was also observed (Fig. 5 and 6). This typical course of morphological transformation was noticed in all HT–100–A (116 days *in vitro*), HT–100–B (97 or 98 days *in vitro*), and HT–10–A (124 days *in vitro*) (Fig. 4). The doubling time, estimated on the 122nd day after the treatment, was 21·8 ± 1·50 hours in HT–100–A, 28·4 ± 2·04 hours in HT–10–A, and 27·9 ± 1·09 hours in the control, and 22·4 ± 1·60 hours in HT–100–A, 20·5 ± 1·10 hours in HT–10–A and 31·2 ± 2·0 hours in the control examined on the 271st day after treatment (Table I). As shown in Table I and Fig. 7, the plating efficiency of the treated cells was markedly high in the transformation stage (122 days after treatment). Over 10% of the treated cells, HT–100–A and HT—10–A, formed colonies, while only 0·5% of the control cells formed colonies at comparative times.

TABLE I.—*Some Biological Features of Hamster Lung Fibroblastic Cells 122 Days and 271 Days after Tar Treatment (HT-A Strain)*

| | Doubling time (hr) | Colony formation rate (%) | Chromosome mode | No. of variation |
|---|---|---|---|---|
| Control | 27·9±1·09 | 0·5±0·5 | 44 | 42–96 |
| (HA-strain) | 31·2±2·02 | — | 42 | 36–121 |
| AT–10–A | 28·4±2·04 | 10·8±4·96 | 44 | 43–112 |
| | 20·5±1·10 | — | 44 | 41·92 |
| AT–100–A | 21·8±1·50 | 16·0±4·0 | 45 | 41–136 |
| | 22·4±1·60 | — | 45 | 40–90 |

upper: After 121 days
lower: After 271 days

*Chromosome studies*

The chromosome number and constitution of transformed cell lines (HT–100–A and HT–10–A) were studied and compared with those of untreated cells on the 122nd and 270th day *in vitro*. As illustrated in Fig. 8, the modal chromosome number of untreated cells 122 days after the treatment was 44, with a fairly large variation between 42 and 96. On the 271st day of culture, the modal chromosome number was 42 with a fairly sharp secondary peak at 44. The control cells, at the late period, did not show a normal diploid constitution, *i.e.* in the chromosome complement of modal cells of the untreated cultures there was monosomy of No. 6 and 13, trisomy of No. 19, and complete absence of No. 7, 9 and 15, with addition of five small extra chromosomes (Fig. 9).

The treated cell strain, HT–100–A, showed a modal chromosome number of 45 with a rather limited variation of 40 to over 70 on the 122nd and 271st day after treatment. Modal cells of the treated HT–100–A on the 271st day *in vitro* displayed trisomy of No. 19, monosomy of No. 2 and 15, and two extra chromosomes (Fig. 10). The cell strain, HT–10–A, showed a modal chromosome number of 44 with rather limited variation of number from cell to cell on both 122nd and

FIG. 8.—Distribution of chromosome number in cells treated with 100 or 10 μg./ml. of tar and untreated controls 122 and 271 days after the treatment.

271st day *in vitro*. This corresponds to pseudodiploid range. Modal cells of HT–10–A 271 days *in vitro* consisted of monosomy of No. 1, absence of No. 2, probably one extra chromosome, and two extra chromosomes between No. 11 and 13. Chromosome No. 1 had a marked secondary constriction in its long arm (Fig. 11). There were no marker chromosomes in control and HT–100–A strains.

*Transplantation test*

Of HT–100–A, HT–10–A, and control cells were periodically transplanted into the cheek pouch of young golden hamsters from the 30th to the 191st day after the tar-treatment, at about one-month intervals. At 270 days after the treatment, hamsters were injected with $1 \times 10^4$ to $10^7$ cells. The results of serial transplantation are shown in Table II. No tumour developed in hamsters that received cells from control cultures, and no nodule was detected in the animals inoculated with HT–100–A cells between 30 and 120 days after the tar-treatment. When $1 \times 10^6$

TABLE II.—*Transplantation Rate (Inoculum Size* $1 \times 10^6$*/hamster) Tar-treated Cells (HT–A Strain)*

| Days after treatment | ~120 | 121 | 160 | 191 |
|---|---|---|---|---|
| Control . | . 0/9 | . 0/1 | . 0/3 | . 0/2 |
| HT–10–A | . 0/13 | . 0/2 | . 1/3 | . 0/2 |
| HT–100–A | . 0/11 | . 1/2 | . 3/3 | . 3/3 |
| | | | (2) | (1) |

( ) Number of animals that died.

FIG. 9.—Chromosome analysis from modal cell of control (271 days *in vitro*).

cells cultured 121 days *in vitro* were transplanted to two animals, a nodule was observed in each of the recipients 1 week after the transplantation. The nodule gradually regressed in one animal and finally disappeared while the other grew progressively. HT–100–A cells, on the 160th and the 191st day after treatment, grew in the pouches of all 6 hamsters inoculated (Fig. 12). One-half of the hamsters inoculated died from the tumour and the remainder were killed for histological examination. HT–10–A cells did not grow in the pouch of hamsters except in one animal, which has been inoculated with $1 \times 10^6$ cells of HT–10–A after 160 days *in vitro*, noticed on the 35th day after the inoculation (Fig. 13 and Table II).

The cells from HT–10–B and control of B strain did not grow in hamster pouches during this study, but the cells from HT–100–B strain produced tumours in the inoculated part of the animals 95 days after the tar-treatment. Three to six animals died from tumour and others were killed for histological study. The results obtained from HT–B strain was almost the same as those from HT–A strain (Table III). Histologically these tumours consisted of atypical cells, spindle to round in shape, with rather rich cytoplasm. Marked pleomorphism was observed in tumour cells. Tumour cells infiltrated into cheek pouch of a hamster in some cases. These tumours were diagnosed as fibrosarcoma (Fig. 14).

The results of the HT–A strain cell transplantation 270 days after the tar treatment are shown in Table IV. All the animals were killed and examined for

FIG. 10.—Chromosome analysis from modal cell of HT-100-A 271 days after the treatment.

TABLE III.—*Transplantation Rate* (*Inoculum Size* $1 \times 10^6$/*hamster*) *of Tar-treated Cells* (*HT-B Strain*)

| Days after treatment | ~90 | 100 | 160 |
|---|---|---|---|
| Control . | 0/11 . | 0/2 . | 0/2 |
| HT-10-B . | 0/9 . | 0/3 . | 0/5 |
| HT-100-B . | 0/9 . | 2/3* . | 3/3 |
| | | (1) | (2) |

( ) Number of animals that died.
* Cells from 95 days *in vitro*.

nodules 48 days after the inoculation. No tumour was observed in animals that received less than $1 \times 10^5$ of control cells, but $1 \times 10^6$ of control cells grew to tumour size in one animal and the tumour remained in the animal for 48 days. Nodules were noticed in the pouch of animals transplanted with $1 \times 10^7$ cells 3 weeks after the transplantation, and they grew gradually and reached a soybean size 48 days after the transfer (Fig. 15). Transfer of $1 \times 10^4$ to $10^6$ treated cells of HT-100 produce nodules in all the recipients, and 2 of the 5 animals inoculated

FIG. 11.—Chromosome analysis of modal cell of HT-10-A, 271 days after the treatment.

with 1 × $10^6$ cells died. After transplantation of 1 × $10^5$ or $10^6$ HT-10-A cells, nodules grew in all the hamster pouches. When 1 × $10^4$ cells were inoculated into each of two animals, a nodule grew in one of them.

TABLE IV.—*Results of Transplantation of HT-100-A Cell 270 Days after the Tar Treatment*

| | Cell no. | Days after treatment: 27 | Days after treatment: 48 | Remark |
|---|---|---|---|---|
| Control | $10^7$ | 2/3 | 2/3 | Soybean size 48th day |
| | $10^6$ | 1/4 | 1/3* | Remain |
| | $10^5$ | 0/3 | 0/3 | |
| HT-10-A | $10^6$ | 2/3 | 3/3 | |
| | $10^5$ | 0/3 | 3/3 | |
| | $10^4$ | 0/3 | 1/2* | |
| HT-100-A | $10^6$ | 5/5 | 3/3 | Two animals died of tumour |
| | $10^5$ | 4/4 | 4/4 | |
| | $10^4$ | 1/2 | 1/2 | |

* Animal died from pneumonia between 27 and 48 days after the inoculation.

### DISCUSSION

These 10 years, there have been many reports on carcinogenesis *in vitro* induced by chemical carcinogenic aromatic hydrocarbons (Berwald and Sachs, 1963 and 1965; Huberman and Sachs, 1966; Dipaolo *et al.*, 1969*a*, 1969*b*) and some report on the effect of cigarette tar *in vitro* (Awa *et al.*, 1961; Leuchtenberger and Leuchtenberger, 1969), especially on the effect of benzo[*a*]pyrene and 3,4-benzo-[*a*]pyrene, which are main carcinogenic substances in tobacco tar to pulmonary tissues *in vitro.*

In the present study, hamster lung fibroblastic cells were transformed into neoplastic cells by treatment with crude tobacco tar. The general course of transformation was the appearance of morphological transformed fusiform cells with a criss-crossing, piled up and random arrangement of cells about 100 days after the treatment. Subsequently, these cells formed tumours in the cheek pouch of young adult hamsters. No morphological transformation was observed in untreated control cells of two culture strains more than 300 days after the cultures were made. Injection of more than $1 \times 10^6$ cells of control HT–A strain into the hamster pouch, produced tumours at the transplanted site. This phenomenon may be due to the fact that some cells in control cultures underwent spontaneous transformation between the 190th and the 270th day after the beginning of experiment and grew to predominate over untreated control cells. At the same period, modal number of chromosome of HT–A strain and control cells changed from 44 to 42. It may be considered that this phenomenon is one of the evidences for spontaneous transformation in control culture.

In the present study, hamster lung cells were transformed into neoplastic cells by treatment with 10 to 100 $\mu$g./ml. of crude tobacco tar but 100 $\mu$g. of tobacco tar contains very small amounts of carcinogenic aromatic hydrocarbons, *i.e.* $6{\cdot}4 \times 10^{-4}$ of benzo[*a*]pyrene, $1{\cdot}6 \times 10^{-4}$ of dibenz[*a,h*]anthracene, $9{\cdot}25 \times 10^{-5}$ $\mu$g. of dibenz[*a,j*]acridine, etc. Previous workers, however, used 10 to 50 $\mu$g./ml. of carcinogenic hydrocarbons for transformation of cultured cells (Berwall and Sachs, 1965; Dipaolo and Donovan, 1967). The total amount of aromatic hydrocarbons of tobacco tar in this study is 1/143 to 1/715 compared with the amount

---

### EXPLANATION OF PLATES

FIG. 1.—Untreated hamster lung fibroblastic cells on 30th day *in vitro*. (×270.) Note: Oriented arrangement of fibroblastic cells.

FIG. 2.—Late stage of control cells. Note: Large, flat cells (between 135 and 140 days *in vitro*). (×270.)

FIG. 3.—The cells 48 hours after treatment with 100 μg./ml. of tar. Note: Pyknosis of nuclei, swelling, disintegration, and destruction of cytoplasm. (×270.)

FIG. 5.—Transformed foci growing at random and producing a dense layer (HT–100–A, 121 days after the treatment). (×270.)

FIG. 6.—Transformed foci of HT–10–A cells 121 days after the treatment. (×270.)

FIG. 7.—Colony formation of HTA–100–A, HTA–10–A, and control cells (122 days after the treatment).

FIG. 12.—Tumour produced in a cheek pouch of a hamster by inoculation of transformed HT–100–A cells (50 days after inoculation with $1 \times 10^6$ cells 160 days after the treatment).

FIG. 13. Tumour produced in a cheek pouch of a hamster by inoculation of transformed HT–10–A cells 160 days after the treatment (71 days after inoculation with $1 \times 10^6$ cells).

FIG. 14.—Histological section of tumour in cheek pouch (the case inoculated with HT–100–A cells). Note: the marked pleomorphism of tumour cells, mainly spindle in shape, and tumour cells with chromatin-rich nucleus and rather broad cytoplasm. (×82·5.)

FIG. 15.—Tumour produced in cheek pouch of a hamster by inoculation of control cells 270 days *in vitro* (48 days after inoculation with $1 \times 10^7$ cells).

1

2

3

Inui and Takayama

5

6

7

Inui and Takayama

12

13

Inui and Takayama

14

15

used for transformation *in vitro* by previous researchers. Moreover, as compared to carcinogenic activity of a strong carcinogen, methylcholanthrene, tobacco tar has about 15 times more carcinogenic activity *in vitro* (Mondal and Heidelberger, 1970). There are two possibilities for this strong carcinogenicity of tobacco tar; (1) tobacco tar contains some strong carcinogenic substances such as nitroso compounds that have not been discovered, and (2) carcinogenicity of tobacco tar may be due to synergic action of carcinogenic hydrocarbons and nitroso compounds in the tar (Boyland and Roe, 1966; Druckrey and Preussmann, 1962). It would benefit to examine the mechanism of tobacco tar carcinogenesis *in vitro* along these two points, and the present experiment demonstrated neoplastic transformation of cells *in vitro* by treatment with tobacco tar for the first time.

This work was supported in part by a Grant-in-Aid from Japan Monopoly Corporation.

### REFERENCES

AWA, A., OHNUKI, Y. AND POMERAT, C. M.—(1961) *Tex. Rep. Biol. Med.*, **19**, 518.
BERWALD, Y. AND SACHS, L.—(1963) *Nature, Lond.*, **200**, 1182.—(1965) *J. natn. Cancer Inst.*, **35**, 641.
BOYLAND, E. AND ROE, F. J. 3.—(1966) ' Carcinogenic Nitrosamines Which may be Present in Cigarette Smoke. Lung Tumours in Animals ', Division of Cancer Res., Perugia, 667.
DIPAOLO, J. A. AND DONOVAN, P.—(1967) *Expl Cell Res.*, **48**, 361.
DIPAOLO, J. A., DONOVAN, P. AND NELSON, R.—(1969*a*) *J. natn. Cancer Inst.*, **42**, 867.
DIPAOLO, J. A., NELSON, R. L. AND DONOVAN, P. L.—(1969*b*) *Science, N.Y.*, **165**, 917.
DRUCKREY, H. AND PREUSSMANN, R.—(1962) *Naturwissenschaften*, **49**, 498.
HUBERMAN, E. AND SACHS, L.—(1966) *Proc. natn. Acad. Sci., U.S.A.*, **56**, 1123.
INUI, N. AND TAKAYAMA, S.—(1971) *Gann*, **62**, 315.
LEUCHTENBERGER, C. AND LEUCHTENBERGER, R.—(1969) *Cancer Res.*, **29**, 862.
MONDAL, S. AND HEIDELBERGER, C.—(1970) *Proc. natn. Acad. Sci., U.S.A.*, **65**, 219.